# Management of a Splenic Artery Aneurysm in the Third Trimester of Pregnancy

**DOI:** 10.1155/2020/8892605

**Published:** 2020-09-19

**Authors:** Oxana Zarudskaya, Madanika Subash, Anita Tamirisa, Nikolina Docheva, Balaji Reddy, Dani Zoorob

**Affiliations:** ^1^University of Toledo, Department of Obstetrics and Gynecology, 2142 N. Cove Blvd., Toledo OH 43606, USA; ^2^University of Toledo, Department of Surgery, 3000 Arlington Ave, Toledo OH 43614, USA

## Abstract

**Background:**

Splenic artery aneurysm (SAA) is a rare but potentially fatal complication associated with high maternal and fetal mortality when occurring during pregnancy.

**Case:**

A 29-year-old G4P3003 at 34 4/7 weeks of gestation was admitted with left upper quadrant pain and newly diagnosed SAA in the hilum. She was scheduled for embolization of the SAA but the night before went into labor. A multidisciplinary team discussion was held, and the patient underwent successful primary low transverse c-section via Pfannenstiel skin incision followed by laparoscopic splenectomy under general anesthesia. She delivered a male newborn with birth weight of 2855 and Apgar score of 8/5. *Summary and Conclusion*. Early diagnosis and management of SAA are key for improved maternal and fetal outcomes. Our case demonstrates that through a multidisciplinary approach and anticipation of the possible clinical scenarios, good outcomes can be achieved.

## 1. Introduction

Splenic artery aneurysms (SAA) are rare and often are incidental findings on ultrasound examination or autopsy [1, 2]. They are four times more common in women than in men [3]. The incidence of SAA ranges from 0.01% to 0.98%; however, the true incidence is unknown as about 95% of the cases are asymptomatic [3, 4]. Only 2 to 3% of these aneurysms rupture, and of those that rupture, about 20 to 40% occur in pregnant women without any comorbidities [4]. Pregnancy is an important risk factor for SAA rupture [1], and its pathogenesis is unclear [2]. SAA rupture is a catastrophic event associated with high maternal mortality (75%) and fetal mortality (95%) [3, 4] and most commonly occurs in the third trimester of pregnancy. Hence, surgical interventions are recommended in the first and second trimesters. However, most cases of SAA are diagnosed following a rupture [2]. Over 100 cases of SAA in pregnancy have been reported in the literature, yet a minority reported both maternal and fetal survival [5]. Because of such a high maternal and fetal mortality, early definitive surgical intervention becomes a cornerstone in management. We present a case of symptomatic SAA in the third trimester of pregnancy and its management successfully by a laparoscopic splenectomy.

## 2. Case Report

**Figure 1:**
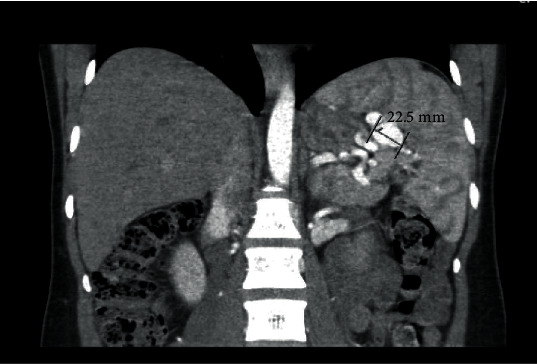
CT abdomen with contrast showing the splenic artery aneurysm (reproduced from Meghan et al. [9], [under the Creative Commons Attribution License/public domain]).

**Figure 2:**
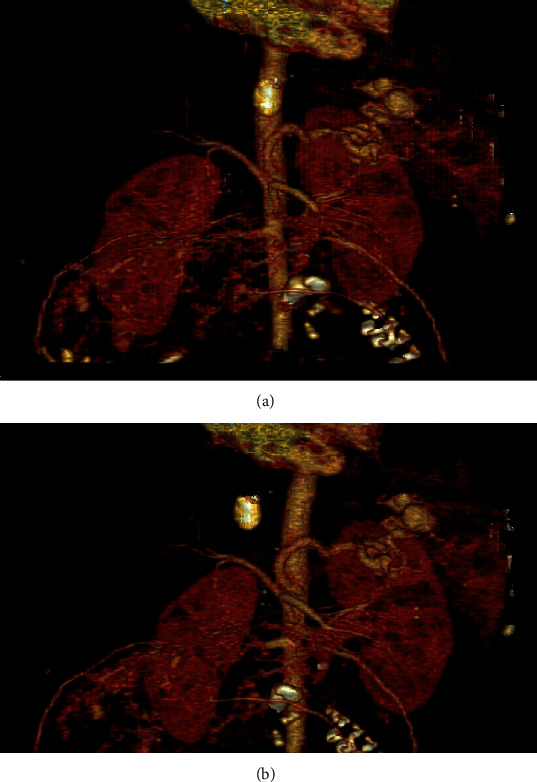
(a) CT angiogram 3-D reconstruction showing the tortuous splenic artery aneurysm (anterior view). (b) CT angiogram 3-D reconstruction showing the tortuous splenic artery aneurysm (posterior view).

A 29-year-old G4P3003 at 34 weeks and 4 days of gestation was transferred from an outside hospital to our tertiary care center with left upper quadrant pain and newly diagnosed splenic artery aneurisms. Initial evaluation was with an abdominal ultrasound followed by CT (Figure 1) and CT angiogram which showed two SAA of 2.3 cm and 1.9 cm in the hilum (Figures 2(a) and 2(b)).

The nature of the pain has been intermittent and present since her last pregnancy four years ago. However, the pain worsened acutely and persisted requiring her to seek emergent care a day prior to her admission. Her past medical history was significant for abnormal pap smears, history of herpes, recent urinary tract infection that was treated, bilateral renal stones, and iron deficiency anemia. The total timeframe between worsening of the pain prompting the diagnosis of the aneurysm and thereafter labor onset followed by delivery was less than 24 hours.

Family history was significant for maternal autoimmune hepatitis and primary biliary cirrhosis.

On examination, vital signs and physical exam were normal except for tenderness in the left upper quadrant on abdominal exam, but without rebound or guarding. Fetal monitoring showed reactive and reassuring tracings, and tocometry revealed irritability without regular contractions. On admission, preterm labor workup was negative (cervical length 3.5 cm by transvaginal ultrasound, cervix 1 cm dilated, 60% effaced, fetal station -3, and negative fetal fibronectin). Also, laboratory studies were performed including liver enzymes, amylase, and lipase that were within normal.

Regarding management, the care of this patient was approached in a multidisciplinary fashion involving the general obstetrics team, maternal-fetal medicine, vascular surgery, general surgery, trauma surgery, interventional radiology, and the neonatal intensive care team. A contingency plan for exploratory laparotomy, neonatal delivery, and control of the maternal hemorrhage was made with the team in case of a SAA rupture. This entitled that the patient was close to a fully staffed OR suite that has the available surgical instruments required, on-call teams being notified, availability of blood products and cell saver for massive transfusion protocol if needed, and space for neonatal resuscitation. The patient was kept nothing by mouth, was typed and crossed, and had 2 IV access sites. Since the patient was stable upon transfer, different management options were discussed in detail. This included delivery with open splenectomy; however, prematurity risks had to be taken into consideration. Therefore, plan was made to attempt to embolize the SAA and allow the pregnancy to progress, taking into account the risk of splenic necrosis and need for future splenectomy. Antenatal corticosteroids were administered. However, due t4o unforeseen circumstances on day 2 of her admission, the interventional radiology suite was not available for the use of elective procedures. During that night, the tracing became category 2, and contractions were noted on the monitor. The patient's cervix was checked, and she was found to be 3 cm dilated. Preterm labor was diagnosed. Vaginal delivery should be ideally avoided in SAA due to the risk of rupture with Valsalva and increased intra-abdominal pressure.

After a brief discussion with the multidisciplinary teams on-call and obtaining patient consent, a decision was made to proceed with a primary low transverse c-section via Pfannenstiel skin incision followed by laparoscopic splenectomy under general anesthesia. Splenic artery aneurism was considered an indication necessitating the avoidance of the second stage of labor (pushing) due to risk of rupture when repetitive Valsalva maneuvers are necessary. Additionally, a persistent category II tracing at the onset of labor was concerning for the fetus being intolerant of labor per se.

**Figure 3:**
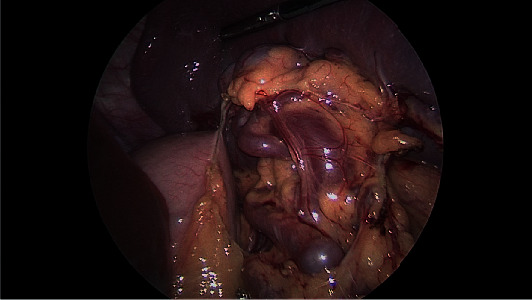
Intraoperative image of the splenic artery aneurysm (reproduced from Meghan et al. [9], [under the Creative Commons Attribution License/public domain]).

Upon entry into the abdomen, no bleeding was noted. The patient delivered a liveborn male fetus at 33 weeks and 5 days with Apgar scores of 8 and 5 at 1 and 5 min, respectively, and birth weight of 2855 grams. The estimated blood loss for the delivery was 400 cc. A decision was made to close the peritoneum upon abdominal closure to help prevent CO_2_ escape during the laparoscopy. Once the caesarean incision was closed, the laparoscopic splenectomy was performed with resection of the splenic artery proximal to the aneurysm after mobilizing it. Multiple splenic aneurysms were noted in the distal third of the splenic artery; one of them was approximately 2 cm proximal to the hilum (Figure 3). Estimated blood loss during the laparoscopic procedure was 10 ml. The patient recovered well postoperatively. She was counseled about the risks of infection with asplenia and was given postsplenectomy vaccines (Meningococci, HiB, and Pneumovax). She was also given intravenous iron injection for her iron deficiency anemia. She was discharged home on postoperative day 3.

## 3. Discussion

SAA is defined as pathological dilation of the splenic artery—more than 1 cm in diameter [3]. They are the most common visceral artery aneurysms [3] and the third most common abdominal aneurysms following infrarenal aortic and iliac artery aneurysms [4]. The risk factors for SAA rupture include pregnancy, multiparity, portal hypertension, atherosclerosis, medial fibrodysplasia, pancreatitis, aneurysm size greater than 2 cm, rapid aneurysmal growth, and liver transplantation [1, 6]. The pathogenesis of SAA is unclear. Increased levels of estrogen, progesterone, and relaxin during pregnancy are associated with structural changes in the arteries including medial degeneration and fragmentation of the elastic fibers. Physiological changes during pregnancy and compression of the aorta and iliac arteries by the gravid uterus are associated with increased blood flow, portal hypertension, and splenic arteriovenous shunting [2, 3, 6]. Diagnosis of SAA is challenging due to its rarity and vague presentation such as nausea, vomiting, and abdominal pain. Routine screening in pregnancy is not warranted. Angiography is the gold standard for diagnosis, and in pregnant women, ultrasound with pulsed Doppler is the preferred imaging modality [3]. Other diagnostic methods include CT, MRI, and endoscopic ultrasound [3, 7]. X-ray can diagnose SAA when the artery is calcified.

Ruptured SAA presents as an acute abdomen with signs and symptoms of hemorrhagic shock. Pain is usually described as sharp, localized to the left upper quadrant or epigastrium and radiating to left shoulder (Kehr's sign) [3]. In pregnant women, it might be misdiagnosed as uterine rupture (in 70% of cases), abruptio placenta, amniotic fluid embolism, or other surgical emergencies such as perforated peptic ulcer disease [3, 4]. Rupture of SAA is usually a rapid process; however, in about 25% of cases, it can occur in two stages. The initial hemorrhage might be contained in the lesser sac by clots blocking the foramen of Winslow. This stage may last for a few hours to days presenting with mild to moderate pain. Prompt diagnosis and treatment at this stage are associated with better maternal and fetal outcomes. The second stage of rupture is associated with bleeding into the abdominal cavity and is catastrophic [3].

Management of SAA should be multidisciplinary and depends on the presentation and characteristics of the aneurysm [3]. Surgical intervention is required for symptomatic SAA regardless of their size, SAA > 2 cm in diameter and any SAA in a pregnant women [8]. Percutaneous angiographic embolization, transcatheter embolization, and laparoscopic ligation or resection are treatment modalities [4]. In pregnant women, any size of SAA is treated surgically as 50% of SAA that rupture during pregnancy are less than 2 cm [3, 4]. In stable pregnant women, minimally invasive surgical techniques such as occlusion, resection, or arterial bypass are performed depending on the location of the aneurysm [4]. About 80% of SAA are located in the distal one-third [3]. Simple ligation can be performed for aneurysms in the proximal one-third due to good collateral circulation. For aneurysms in the middle third, ligation of the proximal and distal artery is performed. Distal third aneurysms require resection and splenectomy.

Management of ruptured SAA in pregnant women mandates resuscitative measures followed by laparotomy for caesarean section prior to resection of the aneurysm and splenectomy [3, 4, 7]. Caesarean section helps in hemodynamic stability and has shown to improve maternal and fetal survival [7]. Mortality rates for management of stable SAA ranges from 0.5% to 1.3% compared to 75% associated with ruptured SAA [4]. Postoperative counseling of asplenic patients about the risk of infection and vaccination against polysaccharide encapsulated bacteria like pneumococci, haemophilus influenza type b, meningococci, and influenza virus should be done [7].

From an educational standpoint, this case report stresses the need for considered SAA as a diagnosis in the differential for abdominal pain during pregnancy. Additionally, this case report can serve as a guide for future management of SAA cases in pregnant patients by offering suggestions to management (laparoscopic and open approaches), guiding with decision making, as well as ensuring multidisciplinary management (such as involving vascular surgery and anesthesia) closely in planning and care. From a safety and logistics standpoint and due to the significant maternal and fetal risks associated if rupture ensues, it is critical that obstetric, surgical, anesthesia, and institutional readiness be assured when caring for such a high-risk patient.

## 4. Conclusion

Splenic artery aneurysm is a rare condition, and its rupture is associated with high maternal and fetal mortality. Early diagnosis and management is key for improved maternal and fetal outcomes. Our case demonstrates that through a multidisciplinary approach and anticipation of the possible clinical scenarios, good outcomes can be achieved. Hence, it is important for the physician to be aware of this condition and keep it in mind as a differential when a pregnant woman presents with an acute abdomen.
